# Case report of Osteopoikilosis in patient with psoriatic arthritis

**DOI:** 10.1002/ccr3.5263

**Published:** 2022-01-12

**Authors:** Ines Cherif, Kaouther Maatallah, Hanene Ferjani, Wafa Triki, Dorra Ben Nessib, Dhia Kaffel, Wafa Hamdi

**Affiliations:** ^1^ Rheumatology department Med Kassab institute of orthopedics Tunisia Faculty of medicine of Tunis University Tunis el Manar Mannouba Tunisia

**Keywords:** Osteopoikilosis, primary infertility, psoriasis, psoriatic arthritis

## Abstract

Osteopoikilosis (OPK) is one of the rare genetic musculoskeletal, non‐inflammatory disorders that we should increase awareness toward. We report a case of a patient diagnosed with psoriatic arthritis with incidental imaging findings of lesions suggestive of osteopoikilosis.

## Abstract 2

Osteopoikilosis(OPK) is a benign, genetic, non‐inflammatory, bone disorder. The importance of acknowledging this disease resides essentially in ruling out other affections, especially malignant ones. OPK has been reported to be associated with various dermatological, cardiac, and endocrine affections. No specific therapy is required other than the treatment of the coexisting pathologies.

## 1 CASE REPORT

A 38 years old female, initially presented to our rheumatology department for inflammatory right hip pain evolving for two years. She had a family and personal history of cutaneous psoriasis and was followed for primary infertility with premature menopause at the age of 37. As for the surgical history, the patient underwent resection of a breast nodule that turned out to be benign. She did not have any other extra‐articular involvements. Clinical examination showed a permanent irreducible flexion of the right hip of 10° with very painful mobilization hindering the examination and a right quadriceps amyotrophy. The left hip was painless to mobilization with limited internal and external rotation as well as a limited abduction. Provocative sacroiliac joint maneuvers were difficult to perform because of the pain. The lumbar spine was painless and not limited. The left shoulder was painful when mobilized but not limited. Subacromial impingement tests and Jobe's test were positive on the left. The examination of the right shoulder was normal.

## 2 BLOOD AND IMAGING EXPLORATIONS

Blood test results showed an inflammatory syndrome with a high Erythrocyte sedimentation rate (ESR) equal to 89 mm/hr, and high C reactive protein (CRP) equal to 13 mg/L, cell blood count (CBC), hepatic, renal assessments were normal. The phosphocalcic assessment did not show any abnormalities aside from a hypovitaminosis D of 5,23 ng/ml (<30 ng/ml).

Biochemical markers of bone remodeling revealed an elevated alkaline phosphatase equal to 137 IU/L (>104 IU/L). Rheumatoid factor was negative.

**FIGURE 1:**
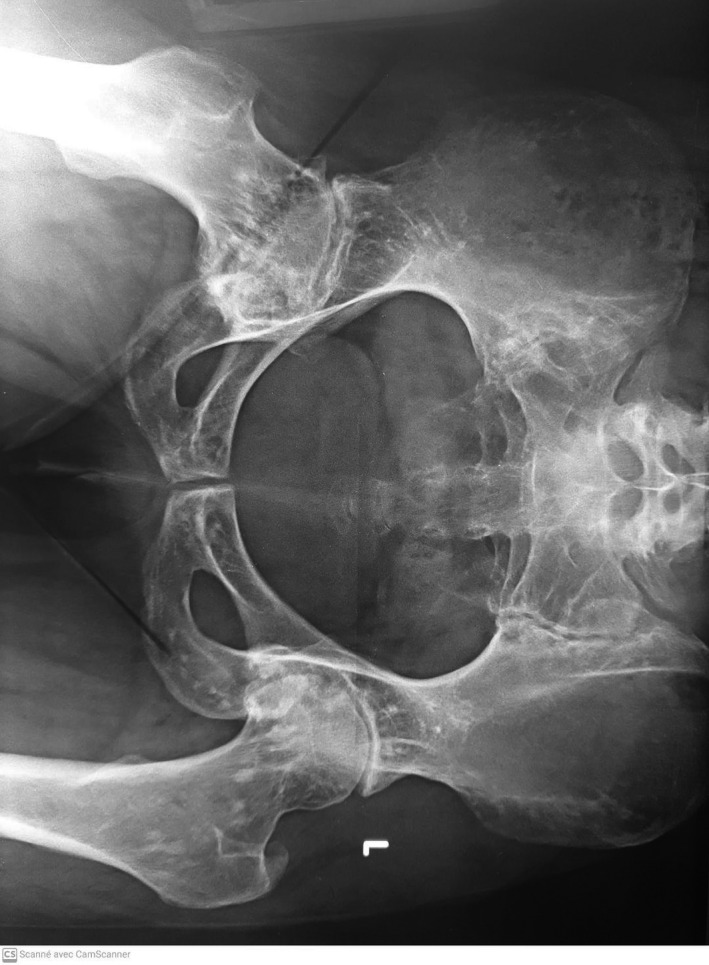
X‐ray of the pelvis demonstrating a Grade 3 right sacroiliitis, a grade 2 left sacroiliitis according to the modified NY criteria, a right destructive coxitis and scattered, symmetric, bilateral, and periarticular sclerotic foci of variable sizes on the pubis, ischium, and ilium as well as on the neck of the femur

**FIGURE 2:**
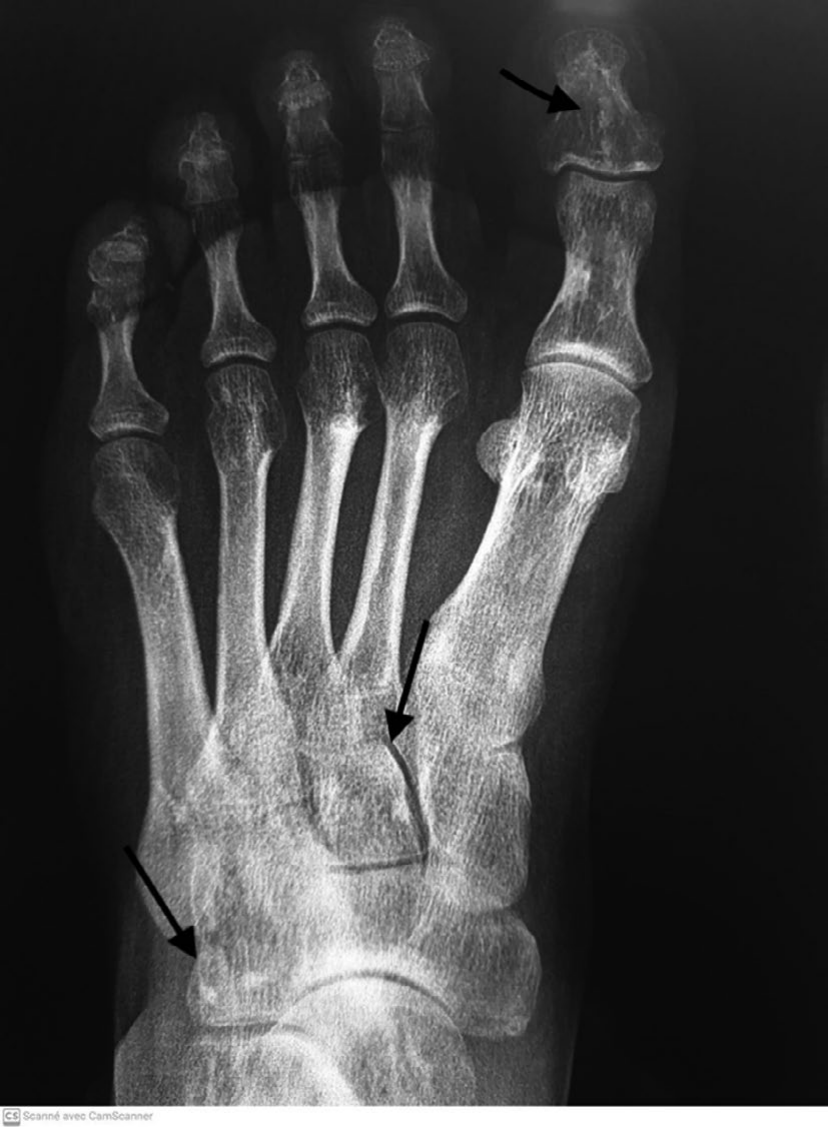
X‐ray of the left foot with well‐limited osteocondensing lesions affecting the tarsals, metatarsal bones, and the hallux’ two phalanges (arrows)

**FIGURE 3:**
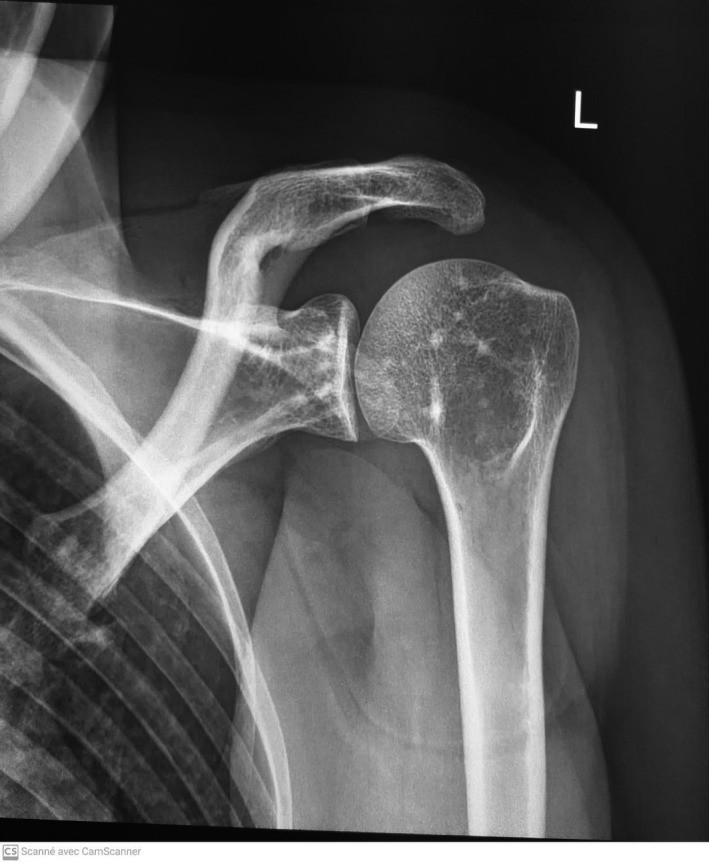
X‐ray of the left shoulder with periarticular sclerotic foci affecting the humeral head and the glenoid with no rupture of the cortical bone

X‐rays of the pelvis, hands, left shoulder, and feet were performed as well as an osteoarticular ultrasound imaging. The ultrasound imaging of the hips showed a destructive arthropathy of the right hip, small effusion without signal on color Doppler, and fatty degeneration of the gluteal muscles. X‐ray showed, however, a right coxitis, a bilateral sacroiliitis, grade 3 on the right, and grade 2 on the left, according to the modified NY criteria.^13^ Besides, X‐rays of the pelvis, left shoulder and feet revealed, incidentally, scattered periarticular sclerotic foci of variable sizes (Figures 1, 2 and 3) suggestive of osteopoikilosis.

The diagnosis of Psoriatic arthritis was retained according to CASPAR criteria,^14^ and the patient was put on sulfasalazine 2 g per day gradually, due to her willingness to conceive and underwent physical therapy. The patient also had an intra‐articular hip injection of Hexatrione.

## 3 DISCUSSION

Osteopoikilosis or “spotted bone disease” is a benign condition, originally described in 1905 by Stieda as "a circumscribed condensing of the bone in the region of the substantia spongiosa".^2^

It is characterized by sclerotic bone lesions most commonly involving the hands, feet, pelvis, and the ends of long bones. They typically spare the skull, ribs, and vertebrae.^3^

In our case, X‐rays of the pelvis, shoulders, and feet showed lesions suggestive of osteopoikilosis, whereas radiographs of the spine and hands showed no such images.

The lesions, when occurring in the pelvis, can sometimes hide or mimic a sacroiliitis presenting a series of radiological diagnostic challenges.^4^

Lesions are generally found incidentally on imaging studies done for other purposes manifesting in symmetric, oval, and periarticular sclerotic bone areas. This was the case for our patient.

Research has shown that the main defect lies in germline mutations in LEMD3, which encodes the inner nuclear membrane protein MAN1, which in turn appears to play a role in the signaling of bone morphogenetic proteins.^5^ A familial tendency exists and suggests an autosomal dominant inheritance,^2^ although our patient did not report similar cases in her family, and family members were not examined in the present study.

In most cases, osteopoikilosis is asymptomatic, although joint pain and joint effusion may be observed in 20% of patients.^3^ The complaints of our patient can, however, be explained by her inflammatory rheumatic disease causing the right coxitis.

Several differential diagnoses may arise, the most urgent ones being osteoblastic metastases and primary bone tumors. Lesions may also sometimes suggest mastocytosis, tuberous sclerosis, synovial chondromatosis, and melorheostosis. The latter is a mesenchymal dysplasia involving a “flowing” pattern of hyperostoses of the cortex of tubular bones and has also been linked to LEMD3 mutations.^1^ It is, though, typically unilateral and monostotic.

Pertaining to malignant lesions, asymmetrical involvement with lesions of varying sizes, preferably located in the axial skeleton, has been more frequently reported. It is usually associated with osseous destruction and positive scintigraphy findings.^6^ A positive bone scan, however, does not rule out the diagnosis of osteopoikilosis.^3^

Our patient did not benefit from scintigraphy because the radiological images were quite suggestive of osteopoikilosis: bilateral, symmetrical, well‐limited, and periarticular. Besides, our patient had a good general state, no bone pain, and did not report any weight loss. Her CBC and the serum protein electrophoresis (SPEP) test showed no abnormalities.

Osteopoikilosis has been reported to be associated with various skeletal and dermatologic disorders such as Klippel‐Feil syndrome,^7^ the Buschke‐Ollendorff syndrome (BOS),^3^ synovial chondromatosis and chondrosarcoma^8^ raising a legitimate question about the necessity for a follow‐up.^3^

OPK has also been described in association with renal and heart malformations, and endocrine disorders.^9^

The number of reported cases in association with rheumatic diseases is expanding^8^ and includes association with rheumatoid arthritis, juvenile arthritis, spondyloarthritis, and psoriatic arthritis.^10^, ^11^, ^12^ Practitioners are therefore wondering whether OPK might be a risk factor or an association with other diseases.

In the present case, the patient has a medical history of both primary infertility because of primary ovarian insufficiency (POI) and psoriatic arthritis. To our knowledge, no case of osteopoikilosis with primary infertility has been reported yet.

## 4 CONCLUSION

OPK is a benign genetic musculoskeletal disorder. The interest in acknowledging this disease lies in the importance of ruling out the differential diagnoses, especially neoplastic causes. This condition seems to be more and more associated with inflammatory rheumatic diseases, raising a legitimate question on whether it might be a potential association.

Some authors suggest a follow‐up, due to a case report of OPK associated with chondrosarcoma. In most cases, the authors do not recommend any routine follow‐up visits or studies. No specific therapy is required other than the treatment of the coexisting pathologies.

## CONFLICT OF INTEREST

None.

## AUTHOR CONTRIBUTIONS

Dr. Ines Cherif, corresponding author: Writing‐Original draft preparation. Dr. Kaouther Maatallah: Conceptualization, Methodology, and Software. Dr. Hanene Ferjani Dr. Wafa Triki, and Dr. Dorra Ben Nessib: Visualization and Investigation. Dr. Dhia Kaffel and Dr. Wafa Hamdi: Supervision.

## PATIENT CONSENT

As stated before, the patient gave her informed consent prior to her inclusion in the report.

## CONSENT

Written informed consent was obtained from the patient to publish this report in accordance with the journal's patient consent policy.
